# Nosocomial spread of OXA-232-producing *Klebsiella pneumoniae* ST15 in a teaching hospital, Shanghai, China

**DOI:** 10.1186/s12866-019-1609-1

**Published:** 2019-10-28

**Authors:** Xin Li, Wei Ma, Qin Qin, Shanrong Liu, Liyan Ye, Jiyong Yang, Boan Li

**Affiliations:** 1 Center for Clinical Laboratory Medicine, Chinese PLA General Hospital, Beijing, 100853 China; 2 Department of Laboratory Diagnosis, Changhai Hospital, Navy Medical University, Shanghai, 200433 China; 3 Center for Clinical Laboratory, the 302 Hospital of Chinese PLA, Beijing, 100039 China

**Keywords:** Outbreak, *Klebsiella pneumoniae*, OXA-232, Sequence type

## Abstract

**Background:**

The spread and outbreak of *Enterobacteriaceae* producing OXA-48-like carbapenemases have become more and more prevalent in China.

**Results:**

A total of 62 non-duplicated OXA-232-producing *K. pneumoniae* (OXA232Kp) were isolated between 2015 and 2017. An outbreak of OXA232Kp was observed in burn ICU. The 62 OXA232Kp isolates were all belongs to ST15 and categorized into two PFGE types (A and B). Type A was dominated of the isolates, which contained 61 clinical isolates and divided into 10 subtypes (A1-A10). In addition, most of OXA232Kp strains exhibited low-level carbapenems resistance. All strains carried a 6141 bp ColKP3 plasmid harboring the *bla*_OXA-232_ gene which is highly homologous to other *bla*_OXA-232_-bearing plasmids involved in other studies in eastern China.

**Conclusions:**

In this study, clone transmission of OXA232Kp ST15was observed. Highly significant homology among the *bla*_OXA-232_-bearing plasmids indicated the important role of the 6.1 kb ColE-like plasmid on the prevalence of *bla*_OXA-232_ gene in China.

## Background

The most alarming resistance trends are those observed for *Enterobacteriaceae*, and of particular concern has been the emergence of resistance to carbapenem antimicrobial drugs among *Enterobacteriaceae*. Carbapenem resistance in *Enterobacteriaceae* is usually attributed to the production of various carbapenemases, a set of enzymes that lead to the hydrolysis of beta-lactams [1]. OXA-48, a novel class D carbapenemase, was identified in a clinical *Klebsiella pneumoniae* isolate in 2004 [2]. Since then, OXA-48-producing *Enterobacteriaceae* has been reported worldwide [3]. Up until now, OXA-48 and its several variants have been identified in *Enterobacteriaceae*. These variants are one to five amino acid substitutions differs from OXA-48 [3, 4]. Most of OXA-48-type carbapenemases hydrolyze carbapenems slightly, while some OXA-48-like variants (such as OXA-163, OXA-247 and OXA-405) only hydrolyze expanded-spectrum cephalosporins but not carbapenems [4]. However, associated with the impaired permeability or the production of extended-spectrum β-lactamases, some OXA-48-like enzymes can lead to high-level carbapenems resistance [5].

OXA-48 was mainly detected in *K. pneumoniae*. Diverse sequence types (STs) of dominant OXA-48-producing *K. pneumoniae* have been identified in outbreaks or solitary case reports worldwide [3]. ST11 and ST116 were present in Taiwan, China [6]. An outbreak of OXA-48-producing *K. pneumoniae* ST147 and ST383 has been reported [7], while other clones, such as ST37 and ST307, have also been identified sporadically [8]. The *bla*_OXA-48_ gene is mainly found on an IncL/M-type self-transferable plasmid of approximately 62 kb, and flanked by two IS*1999* elements to form a functional composite transposon Tn*1999*, which does not carry any other antibiotic resistance gene [2, 5]. In addition, several *bla*_OXA-48_-bearing Tn*1999*-like transposon derivatives (Tn*1999.2* to Tn*1999.5*) were also discovered [3].

OXA-232, an OXA-48-like variant, was identified in France from a *K. pneumoniae* isolate in 2013. OXA-232 differs one (at Arg214Ser) and five amino acid substitutions from OXA-181 and was considered to be a mutant derivative of OXA-181 and not from OXA-48 [9]. Similar to OXA-48, OXA-232 has a reduced ability to hydrolyze carbapenems but exhibits higher hydrolysis activity against penicillins [9]. Since then, OXA-232-producing *K. pneumoniae* (OXA232Kp) had been identified worldwide. These OXA232Kp isolates belong to ST14, ST15, ST16, ST17, ST147, ST231, ST307, and ST395 [10–18]. Almost all OXA232Kp isolates had plasmids with more than 99% sequence identity to pOXA-232, and had a size of 6.1-kb and carried the ColE-type backbone [9]. All these ColE-type plasmids carried the mobilization system (MobA-D), a replication gene (*repA*), truncated parts of erythromycin esterase (Δ*ereA*) and the transcriptional regulator (Δ*lysR*) [19]. The sequence of pOXA-232 was identical to that of plasmid pKP3-A bearing the *bla*_OXA-181_ gene [20], except for almost entire deletion of the IS*Ecp1* transposase gene in pOXA-232.

OXA232Kp has been reported in eastern China. All these OXA232Kp isolates belong to ST15 and carry a 6.1-kp ColKP3 plasmid bearing the *bla*_OXA-232_ gene [21–23]. In this study, we reported a nosocomial outbreak of OXA232Kp in a teaching hospital in Shanghai involved with 61 patients. The phenotypic and genotypic characteristics of OXA232Kp isolates were analyzed.

## Methods

### Bacterial strains

All *K. pneumoniae* isolates recovered from various clinical specimens between 2015 and 2017 at Changhai hospital in Shanghai, China were collected and further identified by VITEK® MS (bioMérieux SA, Marcy-l’Etoile, France). The common carbapenemase genes including *bla*_KPC_, *bla*_NDM_, *bla*_OXA-48_ and *bla*_IMP_ from isolates that exhibited non-susceptibility to carbapenems were screened by PCR amplification, as previously described [24]. *E. coli* ATCC 25922 was used as the quality control strain for antimicrobial susceptibility test. *Salmonella* ser. Braenderup strain (H9812) was used as a reference standard for PFGE.

### Antimicrobial susceptibility test

The MICs of cefotaxime (CTX), ceftazidime (CAZ), piperacillin-tazobactam (TZP), imipenem (IMP), meropenem (MEM), ertapenem (ETP), amikacin (AK), ciprofloxacin (CIP), tigecycline (TGC), sulfamethoxazole/trimethoprim (SXT) and polymyxin B (PB) were measured by broth microdilution method using Biofosun® Gram-negative panels (Biofosun Biotech, Co., Ltd., Shanghai, China). Results were interpreted according to the interpretive standards of the Clinical Laboratory Standards Institute [25].

### PFGE and MLST

PFGE with restriction enzyme *Xba*I was performed for all OXA232Kp isolates as previously described [26]. The PFGE patterns were analyzed by BioNumerics 7.6 software (Applied Maths NV, Sint-Martens-Latem, Belgium) using the dice similarity coefficient. Strains possessing ≥85% genetic similarity or fewer than four fragment differences in PFGE profiles were considered as the same clone (type). Strains (types) with pattern that differ from the original pattern by ≤3 fragments differences were considered to be a subtype of the outbreak strain [27]. MLST was carried out according to the protocols provided on the MLST website (http://www.pasteur.fr/recherche/genopole/PF8/mlst/Kpneumoniae).

### Resistance genes analysis

Genome DNA was extracted from the clinical OXA232Kp isolates using the DNeasy® UltraClean® Microbial Kit (QIAGEN GmbH, 40,724 Hilden, Germany). Genome sequencing was performed with a paired-end library with an average insert size of 350 bp on a HiSeq X Ten sequencer (Illumina, CA, USA). Then, de novo assembly of the filtered reads was performed with SPAdes 3.12 (http://cab.spbu.ru/software/spades/). Draft genome was assembled into scaffold. Acquired antimicrobial resistance genes and plasmid replicons were identified using the ResFinder and PlasmidFinder software available from the Center for Genomic Epidemiology (http://genomicepidemiology.org/).

## Results

### Emergence and outbreak of OXA232Kp

**Fig. 1:**
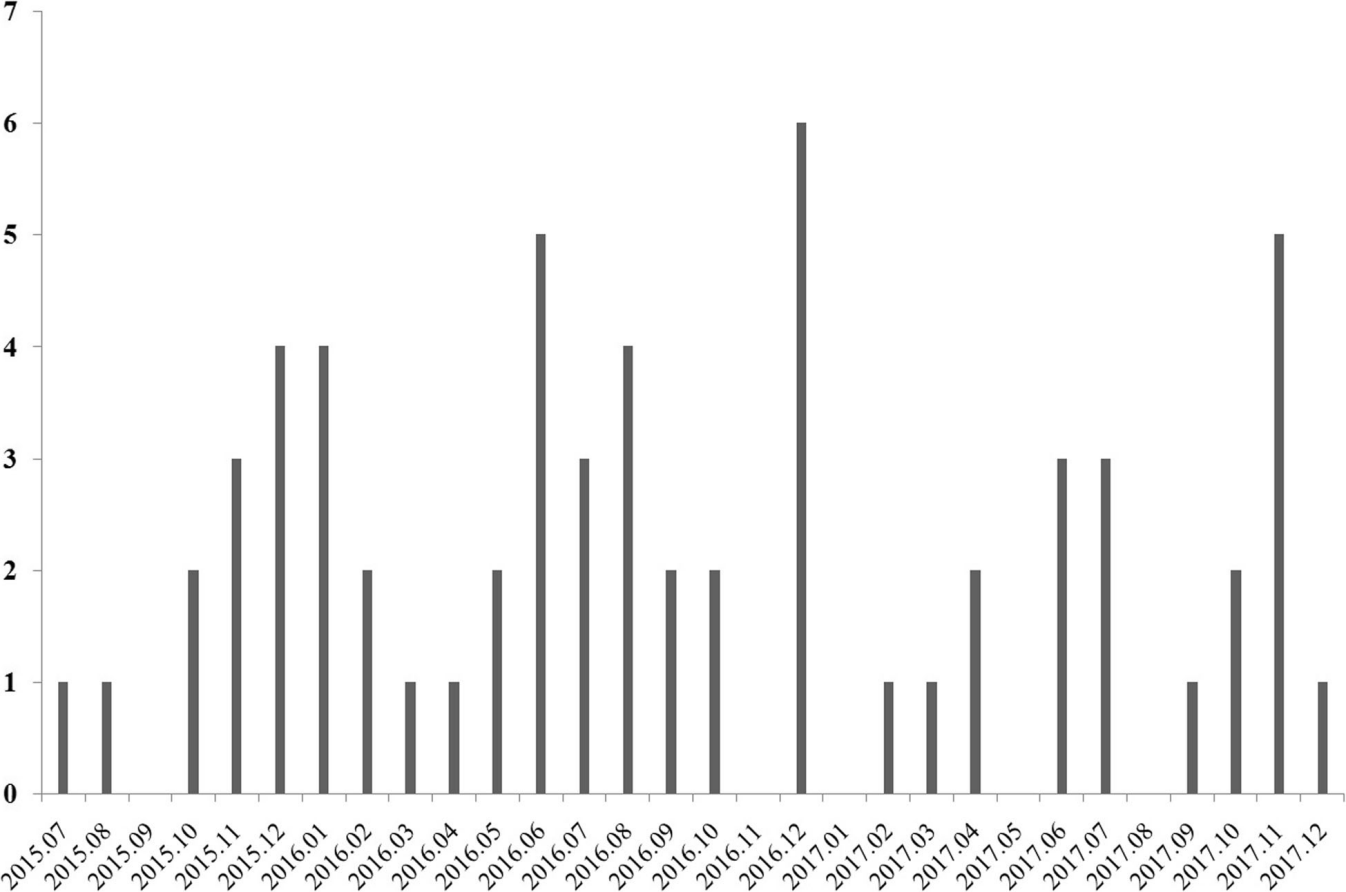
The distribution of the OXA-232-producing *K. pneumoniae* isolates

A total of 141 non-duplicated *K. pneumoniae* isolates that exhibited non-susceptibility to carbapenems were recovered from various clinical specimens between 2015 and 2017. Among these isolates, 62 (43.9%) were OXA232Kp, while 60 (42.6%) produced KPC. Among 62 strains of OXA232Kp, 31 (50%) were recovered from sputum, 11, 11 and 5 isolates were recovered from wound secretion, urine and drainage sample, respectively. In July 2015, OXA232Kp first appeared at the burn ICU, followed by an outbreak in the hospital (Fig. 1). Among 62 patients with OXA232Kp isolates, 7 patients died, and 56 patients (90.3%) showed improvement and discharged.

### PFGE and MLST analysis

The 62 OXA232Kp isolates were categorized into two PFGE types (A and B). The majority of the isolates belonged to types A which contained 61 clinical isolates and was divided into ten subtypes (A1-A10), while types B was detected in only one isolate (Table 1). In July 2015, type A2 of OXA232Kp strain emerged in the hospital. This followed by an outbreak in the burn ICU in the next few months. Meanwhile, new OXA232Kp subtypes (A3-A10) emerged and were found spread to burn ICU other wards (Fig. 1). MLST was performed in one representative of each PFGE types and subtypes. All strains belonged to ST15.

**Table 1:** Phenotypic and genotypic characteristics of OXA-232-producing *K. pneumoniae* isolates

PFGE type	MLST type	Isolates number	Resistance determinants	Minimal inhibitory concentration (mg/L)										
				CTX	CAZ	PZT	IPM	MEM	ETP	AK	TGC	PB	SXT	CIP
A1	ST 15	36	*bla*_CTX-M-15_, *bla*_SHV-28_, *bla*_TEM-1B_, *aac(6′)-Ib-cr*, *qnrB66*, *oqxAB*, *fosA*, *rmtF*, *sul2*	> 64	> 32	> 256	2	2	16	> 128	≤0.5	1	> 8	> 16
A2	ST 15	3	*bla*_CTX-M-15_, *bla*_SHV-28_, *bla*_TEM-1B_, *aac(6′)-Ib-cr*, *qnrB66*, *oqxAB*, *fosA*, *rmtF*, *sul2*	> 64	> 32	> 256	32	32	128	> 128	≤0.5	1	> 8	> 16
A3	ST 15	9	*bla*_CTX-M-15_, *bla*_SHV-28_, *bla*_TEM-1B_, *aac(6′)-Ib-cr*, *qnrB66*, *oqxAB*, *fosA*, *rmtF*, *sul2*	> 64	> 32	> 256	1	2	16	> 128	≤0.5	1	> 8	> 16
A4	ST 15	1	*bla*_CTX-M-15_, *bla*_SHV-28_, *bla*_TEM-1B_, *aac(6′)-Ib-cr*, *qnrB66*, *oqxAB*, *fosA*, *rmtF*, *sul2*	> 64	> 32	> 256	1	2	16	> 128	≤0.5	1	> 8	> 16
A5	ST 15	5	*bla*_CTX-M-15_, *bla*_SHV-28_, *bla*_TEM-1B_, *aac(6′)-Ib-cr*, *qnrB66*, *oqxAB*, *fosA*, *rmtF*, *sul2*	> 64	> 32	> 256	1	4	16	> 128	≤0.5	1	> 8	> 16
A6	ST 15	1	*bla*_CTX-M-15_, *bla*_SHV-28_, *bla*_TEM-1B_, *aac(6′)-Ib-cr*, *qnrB66*, *oqxAB*, *fosA*, *rmtF*, *sul2*	> 64	> 32	> 256	1	4	64	> 128	≤0.5	1	> 8	> 16
A7	ST 15	3	*bla*_CTX-M-15_, *bla*_SHV-28_, *bla*_TEM-1B_, *aac(6′)-Ib-cr*, *qnrB66*, *oqxAB*, *fosA*, *rmtF*, *sul2*	> 64	> 32	> 256	4	8	32	> 128	≤0.5	1	> 8	> 16
A8	ST 15	1	*bla*_CTX-M-15_, *bla*_SHV-28_, *bla*_TEM-1B_, *aac(6′)-Ib-cr*, *qnrB66*, *oqxAB*, *fosA*, *rmtF*, *sul2*	> 64	> 32	> 256	1	2	16	> 128	≤0.5	1	> 8	> 16
A9	ST 15	1	*bla*_CTX-M-15_, *bla*_SHV-28_, *bla*_TEM-1B_, *aac(6′)-Ib-cr*, *qnrB66*, *oqxAB*, *fosA*, *rmtF*, *sul2*	> 64	> 32	> 256	64	64	256	> 128	≤0.5	1	> 8	> 16
A10	ST 15	1	*bla*_CTX-M-15_, *bla*_SHV-28_, *bla*_TEM-1B_, *aac(6′)-Ib-cr*, *qnrB66*, *oqxAB*, *fosA*, *rmtF*, *sul2*	> 64	> 32	> 256	2	4	32	> 128	≤0.5	1	> 8	> 16
B	ST 15	1	*bla*_CTX-M-15_, *bla*_SHV-28_, *bla*_TEM-1B_, *bla*_PER4_, *aac(6′)-Ib-cr*, *qnrB1*, *oqxAB*,*rmtF*, *fosA*	> 64	> 32	> 256	1	2	16	> 128	≤0.5	1	> 8	> 16

**CTX:** cefotaxime, CAZ: Ceftazidime, **PZT:** piperacillin-tazobactam, **IMP:** imipenem, **MEM:** meropenem, **ETP:** ertapenem, **AK:** amikacin, **TGC**: tigecycline, **PB**: polymyxin B, **SXT**: sulfamethoxazole/trimethoprim, **CIP**: ciprofloxacin

### Antimicrobial susceptibilities

The antimicrobial susceptibility patterns were listed in Table 1. All strains presented resistance to third generation cephalosporins and their enzyme inhibitors mixture (CTX, CAZ and TZP), and exhibited heterogeneous carbapenem resistance patterns. However, they were susceptible to tigecycline and polymyxin B.

### Plasmid and resistance determinants

Genome sequencing was performed in one representative strain of each PFGE types and subtypes. All the sequences were uploaded to GenBank with BioProject Accession PRJNA523565. All strains carried *bla*_OXA-232_ gene, which located in a 6141 bp ColKP3 plasmid. In addition, other resistance determinants were also identified (Table 1).

## Discussion

**Fig. 2:**
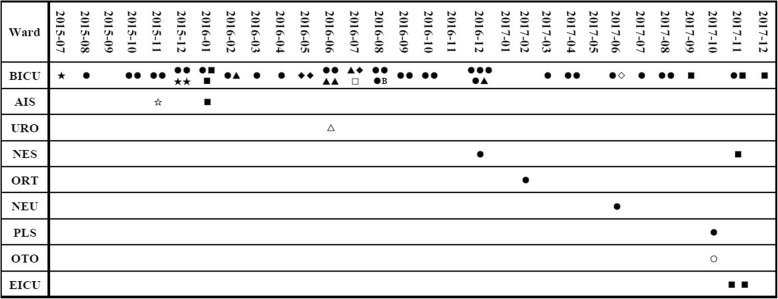
The date, frequency and location of emergence of OXA232Kp strains with different PFGE types The horizontal coordinate shows the date on which OXA232Kp strains were recovered. The wards were listed in the longitudinal coordinate. **BICU**: Burn ICU; **AIS**: Department of anus & intestine surgery; **URO**: Department of Urology; **NES**: Department of Neurosurgery; **ORT**: Department of Orthopedics; **NEU**: Department of Neurology; **PLS**: Department of Plastic surgery; **OTO**: Department of Otolaryngology; **EICU**: Emergency ICU. Clinical isolates with different PFGE types are indicated by various symbols: ●: type A1; ★: type A2; ■: type A3; ○: type A4; **▲**: type A5; ◇: type A6; ◆: type A7; △: type A8; □: type A9; ☆: type A10; B: type B

OXA-232 was first identified in France in 2013 from a *K. pneumoniae* isolate [9]. Since then, OXA232Kp have been discovered worldwide [10–18]. In this study, a nosocomial outbreak of epidemics OXA232Kp involving 61 patients was discovered in a teaching hospital in Shanghai, China. In this hospital, OXA232Kp acted as the main species of carbapenem-resistant *K. pneumoniae* between 2015 and 2017, which 43.9% were OXA-232 positive, while only 42.6% of them produced KPC. This is quite different from data obtained from other part of the world, where production of KPC, OXA-48, and metallo-β-lactamases such as NDM is the main mechanism of carbapenem-resistant *K. pneumoniae* [1]. In this study, OXA232Kp mainly disseminated in the burn ICU (Fig. 2) and appeared to be clonal. At the same time, several outbreak caused by OXA232Kp have been identified in the same area [21–23]. Recently, an outbreak of OXA-48-producing *K. pneumoniae* involving 34 patients at a respiratory ICU has been reported [7]. These results indicated that emergence of *K. pneumoniae* strains producing OXA-48-like carbapenemases have become increasingly frequent in China. It is crucial to recognize the impact of clonal dissemination during the prevalence of these pathogens and strengthen the surveillance and reporting system of carbapenem-resistant *K. pneumoniae* in hospital infection control measures.

In outbreaks of OXA232Kp worldwide, diverse sequence types have been identified, including ST14, ST15, ST16, ST17, ST147, ST231, ST307, and ST395. In Europe and some Asia countries, the majority of OXA232Kp strains were ST14 and ST16 [10–18]. It has been confirmed that *K. pneumoniae* ST15 is the high-risk clones producing OXA-48, KPCs, CTX-M-15 and other ESBLs or carbapenemases. Outbreaks of OXA-48-producing *K. pneumoniae* ST15 have been reported worldwide [28–30]. OXA232Kp ST15 has only sporadically presented in United Kingdom, but there has never been an outbreak [18]. However, all OXA232Kp strains belonged to ST15 in this study (Table 1). Meanwhile, other OXA232Kp strains isolated from eastern China were also ST15 [21–23], suggesting the transmission and spread advantage of this clone in this region, despite the hypovirulence of this clone has been confirmed [23]. Further efforts are required to gain a better understanding about the mechanisms underlying differential prevalence.

According to their case records, 90.3% of patients showed improvement and discharged. Study has shown that OXA232Kp ST15 did not cause lethal infections in immunocompromised patients [23]. Another reason is that the low-level resistance of the stain to carbapenems. The majority of OXA232Kp exhibited low-level resistant or even susceptible to some carbapenems (Table 1). Animal studies and patient outcome data indicated that carbapenems retain meaningful in vitro activity against carbapenemase-producing *K. pneumoniae* isolates with low-level carbapenem resistance [31]. Therefore, timely and effective anti-infective treatment may be an important reason for good outcomes of patients with OXA232Kp infection. However, our data suggest that quinolones and aminoglycosides are no longer suitable for the treatment of infections caused by OXA232Kp (Table 1).Similar to OXA-48, OXA-232 has a reduced ability to hydrolyze carbapenems [9]. Despite the production of CTX-M-15, an enzyme that is considered to mediate high-level carbapenem-resistant phenotypes together with OXA-48-like carbapenemases [5], few OXA232Kp isolates exhibited high-levels of carbapenem resistance (Table 1). Thus, what extent the impacts of ESBLs are on the carbapenem resistance of OXA232Kp is not clear yet. The weak hydrolytic ability of OXA-48-like to carbapenems may lead to an underestimation of the prevalence of clinical isolates producing this group of carbapenemases in China, because most clinical microbiology laboratories only screened the prevalence of carbapenemases among isolates with carbapenem-resistant phenotypes. Changes are needed in clinical microbiology analysis procedures to enhance phenotypic and molecular detection of OXA-48-like carbapenemases.

In this study, all strains carried a 6141 bp ColKP3 plasmid harboring the *bla*_OXA-232_ gene, which was 100% identical to the previously reported *bla*_OXA-232_-bearing plasmid pOXA-232 and pkNICU5, with 100% coverage [9, 21]. Similar plasmid has also been detected in another city in the same region [23]. Except for almost entirely deletion of the IS*Ecp1* transposase gene, the *bla*_OXA-232_-bearing plasmid was identical to that of plasmid pKP3-A bearing the *bla*_OXA-181_ gene [20]. The *bla*_OXA-232_ gene was part of a truncated Tn*2013* transposon. Disrupting the IS*Ecp1* transposase activity, the deletion may have stabilized the *bla*_OXA-232_ gene on the ColE-like plasmid [9]. The high nucleotide sequence homology between the *bla*_OXA-232_-carrying plasmids analyzed in this study and the previously reported plasmid sequences revealed possible sources of the plasmid, and also indicated the significant role of the ColKP3 plasmids on the prevalence of *bla*_OXA-232_ gene in China.

## Conclusion

A clonal dissemination and outbreak of OXA232Kp has been observed, and *K. pneumoniae* ST15 was the predominant clone. Most OXA232Kp strains exhibited low-level resistance to carbapenems. All strains carry a 6141 bp ColKP3 plasmid harboring the *bla*_OXA-232_ gene, which is highly homologous to other *bla*_OXA-232_-bearing plasmids involved in other studies from the same region.

## Data Availability

All the sequences were uploaded to GenBank with BioProject Accession PRJNA523565. All data generated or analysed during this study are included in this published article.

## Abbreviations

AK: amikacin
ATCC: American Type Culture Collection
CAZ: Ceftazidime
CIP: Ciprofloxacin
CLSI: Clinical Laboratory Standards Institute
CTX: Cefotaxime
ETP: Ertapenem
ICU: Intensive Care Unit
IMP: Imipenem
MEM: Meropenem
MS: Mass spectrum
OXA232Kp: OXA-232-producing *K. pneumoniae*
PB: Polymyxin B
PFGE: Pulsed-field gel electrophoresis
ST: Sequence type
SXT: Sulfamethoxazole/trimethoprim
TGC: Tigecycline
TZP: Piperacillin-tazobactam
